# An intelligent fault diagnosis model for bearings with adaptive hyperparameter tuning in multi-condition and limited sample scenarios

**DOI:** 10.1038/s41598-025-92838-4

**Published:** 2025-03-24

**Authors:** Jianqiao Li, Zhihao Huang, Liang Jiang, Yonghong Zhang

**Affiliations:** 1 Faculty of Engineering, Monash University, Clayton, VIC 3800 Australia; 2 School of Automation, Nanjing University of Information Science and Technology, Nanjing, 210044 Jiangsu China; 3 School of Automation, Wuxi University, Wuxi, 214105 Jiangsu China

**Keywords:** Bearing fault diagnosis, CNN, BiLSTM, GWO, Transfer learning, Mechanical engineering, Scientific data

## Abstract

Bearing fault diagnosis under multiple operating conditions is challenging due to the complexity of changing environments and the limited availability of training data. To address these issues, this paper presents an advanced diagnosis method using a hybrid Grey Wolf Algorithm (HGWA)-optimized convolutional neural network (CNN) and Bidirectional long short-term memory (BiLSTM) architecture. The proposed model leverages CNN for extracting spatial features and BiLSTM for capturing temporal dependencies. Through HGWA, hyperparameters are efficiently optimized, achieving 100% diagnostic accuracy across four operating conditions with the CWRU dataset. Additionally, the optimized CNN–BiLSTM model demonstrated high diagnostic accuracy when applied as a pre-trained model in new environments, even with minimal training data. The proposed model not only improves diagnostic performance but also enhances optimization efficiency, achieving faster results within the same time frame. This approach mitigates the challenges of manually tuning neural network hyperparameters and effectively addresses bearing fault diagnosis under constrained sample conditions, representing a meaningful contribution to the field of rolling bearing fault diagnostics.

## Introduction

In complex and demanding mechanical environments, bearings are exposed to various harsh conditions such as vibration, shock, and poor lubrication, which can lead to wear, fatigue, and damage. This not only increases maintenance costs but also poses significant safety risks^1,2^. Therefore, timely and accurate diagnosis and identification of bearing failures are essential to ensure the safe and stable operation of mechanical equipment^3,4^. Rolling bearings play a crucial role in mechanical systems, as their condition has a direct impact on both the efficiency and safety of the equipment’s operation^5–7^. With their outstanding radial load-carrying capacity, rolling bearings are extensively employed in demanding conditions, such as high speeds, heavy loads, and fluctuating loads. However, due to their complex structure and diverse failure modes, rolling bearings are more prone to damage and more difficult to troubleshoot than conventional rotor-bearing systems, such as plain bearings^8^.

In recent years, deep learning (DL) has gained widespread attention and achieved notable advancements in the area of machinery fault diagnosis. An increasing number of scholars are exploring the use of these advanced techniques for detecting bearing faults and predicting the lifespan of bearings, particularly under challenging operating environments^9–11^. For instance, Zhang et al.^12^ developed a technique that fuses vibration and acoustic signal features, which was employed to diagnose faults under variable operating conditions using a multi-input CNN network. This approach has displayed higher accuracy in practical applications compared to algorithms based on unimodal sensors. Zhu et al.^13^ utilized various diagnostic models, including autoencoders (AEs), recurrent neural networks (RNNs), convolutional neural networks (CNNs), and generative adversarial networks (GANs), to tackle the challenges posed by unbalanced and small-sample datasets. Notably, CNNs demonstrated robust feature learning capabilities for fault diagnosis in highly unbalanced data scenarios. Arta et al.^14^ introduced a deep residual network enhanced with an LSTM mechanism for data-driven fault diagnosis, where LSTM’s memory regulation effectively captures temporal information, thereby improving diagnostic accuracy and efficiency. Chen et al.^15^ introduced a CNN model that employs adaptive kernel sizes to automatically capture multi-frequency characteristics from signals. This approach, combined with LSTM networks for classifying fault types based on the extracted features, helps reduce parameter complexity and enhances the overall efficiency of the model. Cui et al.^16^ proposed a method called the triplet attention-enhanced residual tree-inspired decision network (TARTDN) to address the challenge of diagnosing imbalanced bearing faults. By integrating a tree-structured decision network with the Triplet Attention Residual Network (TARN), the model enhances both interpretability and uncertainty quantification, thereby improving decision-making and output recognition. Collectively, these studies have shown promising advancements in fault diagnosis through one-dimensional time-domain signal analysis and by addressing issues of data imbalance and fusion^17^. Keshun et al.^18^ proposed a 3D attention-enhanced hybrid neural network model to overcome the limitations of existing models in capturing bidirectional temporal dependencies within sequential data. This model uses a convolutional neural network (CNN) to extract local features and a bidirectional long short-term memory (BiLSTM) framework to capture long-range dependencies. However, these models often rely on specific parameter settings, which can limit their robustness when applied to new fault types or conditions.

In neural networks, hyperparameters are critical to model performance^19^. Tuning these parameters is a time-consuming and labor-intensive task that relies heavily on empirical methods. Besides, this humdrum work lacks theoretical support, thus limiting the transparency and interpretability of the optimization process. This highlights the need for more systematic and efficient tuning and optimization strategies to make full use of deep learning in fault diagnosis. Fundamentally, hyperparameter optimization in neural networks is a multi-objective optimization problem^20^. Intelligent optimization algorithms possess robust global search capabilities, and they are able to adaptively adjust parameters and strategies to enhance optimization efficiency. Hence, they are particularly effective in tackling complex, dynamic, and uncertain problems, and numerous intelligent optimization algorithms have been employed to address hyperparameter optimization challenges. A fusion intelligence algorithm, combining Differential Evolution (DE) and GWO, is utilized to optimize CNNs^21^, with the goal of improving diagnostic accuracy and noise immunity. Tian et al.^22^ developed a CNN-LSTM model specifically designed for bearing fault diagnosis, which was further optimized using a hybrid particle swarm optimization (HPSO) approach. By capitalizing on HPSO’s strong global optimization abilities, the model dynamically adjusts parameters to address nonlinear and complex multivariate optimization issues, thereby maximizing diagnostic performance. Wang et al.^23^ introduced a novel method, ISSA-LSTM, which integrates the Improved Sparrow Search Algorithm (ISSA) for hyperparameters optimization with LSTM networks, showcasing remarkable prediction accuracy and strong generalization capabilities. Overall, most of the previous studies focused on optimizing model structure and parameter selection to reduce tuning time and improve baseline performance. Yet, they often neglect the fine-tuning of training-specific parameters such as batch size, dropout rate, and learning rate, which are critical for promoting model performance and improving generalization. Despite the importance of model architecture parameters, training parameters should not be neglected as well, and such omissions can lead to suboptimal results and diminish the robustness of the model under different conditions^24–26^. Furthermore, plenty of optimization algorithms tended to fall into local optima and thereby reduce the adaptability of the model, which increases the need for large sample sizes.

In practice, most models typically require a large number of labelled training samples to perform fault diagnosis tasks effectively^27,28^. However, obtaining sufficient fault samples for real-world signal acquisition is often challenging, especially in cases such as rolling bearings, where faults may fail quickly after their occurrence^29^. Typical methods used to address data imbalance are data augmentation, feature learning, and classifier design. Although effective, they may introduce noise, omit features, and still require adjustment in the cases of extreme imbalances^30^. This highlights the need for advanced techniques to enhance fault diagnosis in situations with limited sample availability. To address the challenge of scarce labeled samples in the target domain, Fan et al.^31^ introduced a migration neural network that enables knowledge transfer from related domains, thereby improving diagnostic accuracy in the target domain. Luo et al.^32^ combined a multicore maximum mean deviation migration mechanism with a CNN, training it with unlabeled samples from the target domain, which achieved good performance on experimental data for bearings and gears. Kuang et al.^33^ proposed a novel end-to-end Domain Conditional Joint Adaptation Network, designed to facilitate cross-domain diagnostic knowledge transfer. This approach uses a joint adaptation strategy, enabling domain-level and class-level adaptation through domain adversarial training and dual classifier adversarial training, respectively. Wang et al.^34^ proposed a transfer learning model that integrates a domain adversarial strategy with Wasserstein distance, utilizing source domain data generated by a digital twin (DT) model for training. Experimental results demonstrate that this method outperforms both the hybrid distance-guided adversarial network and the distance-guided domain adversarial network in transfer learning tasks. Qian et al.^35^ developed a Deep Discriminative Transfer Learning Network designed to enhance the diagnosis of faults through effective knowledge transfer across domains. This method displays superior transfer fault diagnosis performance and excels in cross-machine troubleshooting compared to other conventional domain adaptation methods. Ding et al.^36^ developed a novel Deeply Imbalanced Domain Adaptive Migration Learning framework to address the labeling shift caused by class imbalance, achieving fine-grained latent space matching through cost-sensitive learning and classification alignment. While these approaches contribute to reducing the gap between the source and target domains, they are still insufficient in completely addressing the challenge posed by the limited availability of samples in the target domain. Furthermore, many approaches focus on aligning feature distributions but fail to capture the complex, high-dimensional relationships needed for effective fault diagnosis. This can lead to suboptimal performance, especially when the target domain has unique characteristics not represented in the source domain. Therefore, more robust methods are needed to effectively utilize limited labeled data in the target domain while maintaining high diagnostic accuracy.

This paper presents a hybrid fault diagnosis model that combines CNN and BiLSTM with the HGWA optimization algorithm, designed to tackle bearing fault diagnosis across multiple operating conditions with limited training samples. This approach combines CNN and BiLSTM to efficiently extract high-dimensional, time-dependent features from raw acceleration signals. The HGWA algorithm further fine-tunes the training parameters of the pre-existing CNN-BiLSTM model, enhancing its generalization capability and boosting its classification accuracy. By adjusting the pre-trained model using a small set of training samples, the fault diagnosis system becomes capable of adapting to various operating conditions. This approach effectively addresses the challenges of training models and detecting faults in data-limited environments.

In summary, the primary contributions of this paper are as follows.

1. A one-dimensional CNN-BiLSTM network is introduced to effectively extract features and capture temporal dependencies in fault diagnosis, thus improving diagnostic accuracy.
2. The HGWA algorithm was proposed to resolve the inherent problem of local optima and premature convergence in GWO algorithm. This improved optimization algorithm incorporated the crossover and mutation operation of Genetic algorithm (GA) into GWO algorithm, which significantly promote the optimization performance of global search and convergence.
3. The HGWA algorithm was utilized to optimize the training parameters of the CNN-BiLSTM network, leading to a significant reduction in the effort required for parameter tuning. Notably, the optimized model achieved perfect diagnostic accuracy (100%) across four different operating conditions in the CWRU datasets, even with a limited number of training samples.
4. Optimized CNN-BiLSTM networks are capable of achieving high fault diagnosis accuracy with minimal training samples, even when applied to new operating conditions, thus eliminating the need for training from scratch. Certain optimized models have shown remarkable performance in cross-condition fault diagnosis, successfully operating without the need for additional fine-tuning through transfer learning.

This paper is structured as follows: “Related works” section provides an introduction to the fundamental principles of CNN, BiLSTM, and model-based transfer learning. “The hybrid bearing fault diagnosis model with HGWA optimization” section presents the design and optimization process for the one-dimensional CNN-BiLSTM model applied to bearing fault diagnosis. “Diagnosis of bearing faults across various operating conditions” section explores the difficulty of identifying bearing faults under varying conditions with limited training data. It suggests combining optimization models with transfer learning as a strategy to effectively address the issue of data scarcity in practical applications. Experimental results using the CWRU and JNU bearing datasets are presented in “Experimental results” section, highlighting the effectiveness of the proposed approach. Finally, “Conclusion and future work” section concludes the study and outlines potential directions for future research.

## Related works

### Convolutional neural network (CNN)

**Fig. 1:**
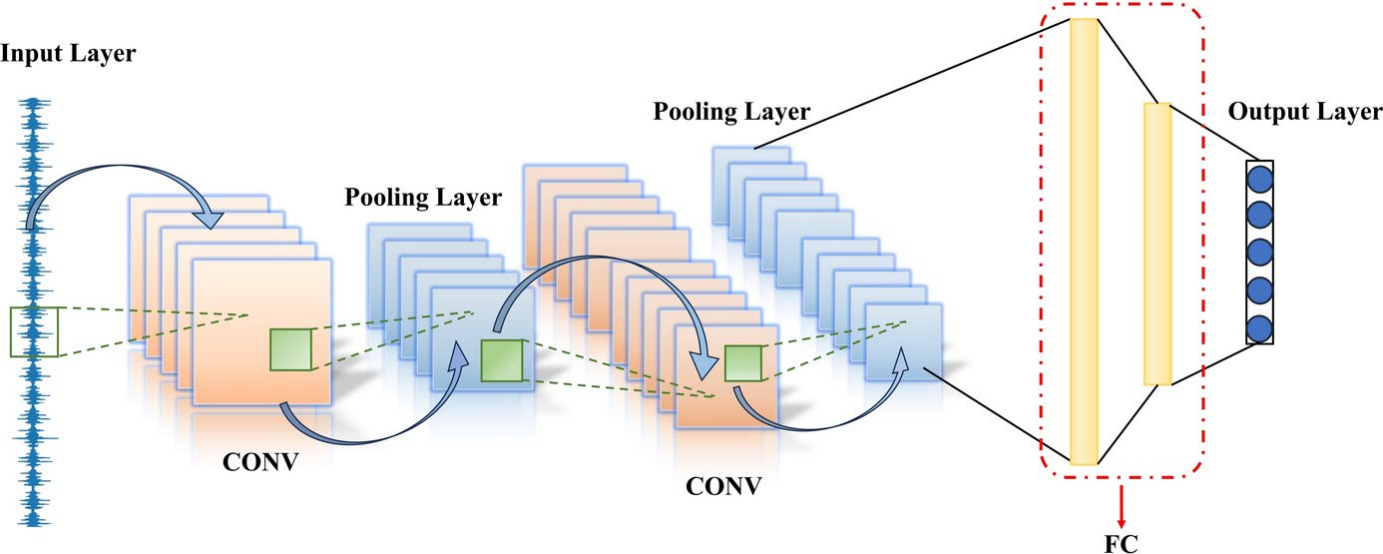
Typical architecture of the CNN.

In recent years, deep learning methods have gained widespread use in the field of rolling bearing fault diagnosis^37,38^. Among these techniques, Convolutional Neural Networks (CNNs) have attracted significant attention due to their remarkable capacity for feature extraction and classification tasks^39^. A typical CNN structure consists of an input layer, several convolutional layers, pooling layers, and an output layer, as illustrated in Fig. 1.

The input layer typically consists of multiple structured arrays. To process data, a raw one-dimensional sample vector can be transformed into a two-dimensional dynamic matrix using the time-lag-shift technique, which then serves as the input for a CNN, enabling efficient feature learning. Convolutional and pooling layers, composed of several feature maps, are fundamental to feature extraction in CNNs and rely on principles of local connectivity and weight sharing. Each neuron in the convolutional layer’s feature maps connects to a specific local patch through a convolutional kernel, where the kernel size matches that of the local patch. The entire feature map is generated by sliding this shared kernel over various local patches in the previous layer’s feature map. Following the convolution, a Rectified Linear Unit (ReLU) activation function is typically applied to enhance the network’s nonlinear representation, as defined below.

1 $$f\left( x \right) = \max \left( {0,x} \right)$$

The pooling layer, often termed the subsampling layer, generally followed the convolutional layer to further compress features within the input feature maps. This operation reduces the network’s size while enhancing the effectiveness of the extracted features. The pooling layer uses statistical methods to derive a representative value for each local patch. Common pooling operations include max pooling and average pooling. Unlike in the convolutional layer, the local patches in the pooling layer generally do not overlap. This leads to the output feature maps being reduced to approximately $1/k^{2}$ of the input size, where $k \times k$ represents the dimensions of the connected local patches. Therefore, the pooling layer is crucial for decreasing the parameter scale in CNNs.

The output layer consists of several fully connected (FC) layers, which integrate the high-level features extracted by the preceding layers for specific regression or classification tasks. These features are flattened into a one-dimensional vector to serve as the input for the output layer.

### Bidirectional long and short-term memory network (BiLSTM)

The LSTM network is a special type of Recurrent Neural Network designed for processing and predicting time series data^40,41^. Unlike traditional RNNs, LSTM overcomes the gradient vanishing and gradient explosion problems encountered when processing long time series by capturing long-term dependencies^42,43^. Nevertheless, LSTM can only process forward sequence information and fails to capture both forward and backward information simultaneously. Lately, BiLSTM networks were specifically developed to resolve the inherent limitation with LSTM. BiLSTM extends the traditional LSTM by incorporating two networks, i.e., one processes forward sequences (from front to back), and the other processes reverse sequences (from back to front)^44^. This bidirectional structure allows BiLSTM to capture both forward and backward information, thereby enhancing the understanding and modeling of sequence data.

**Fig. 2:**
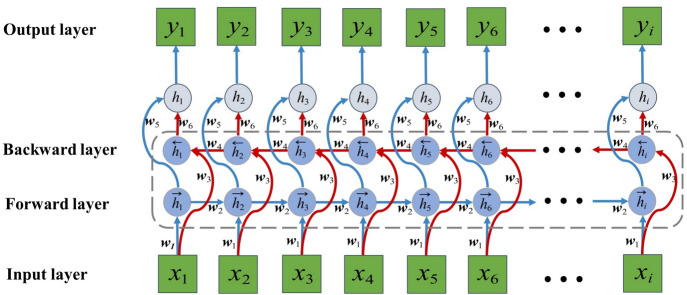
Structure of BiLSTM network.

In Fig. 2, the structure of the BiLSTM network^45^ is illustrated. In this architecture, the forward layer conducted computations step-by-step in a forward direction, recording the outputs of the forward hidden layer at each step. Then, the backward layer processed the data in reverse, storing the outputs of the backward hidden layer accordingly. Finally, the outputs from both layers were integrated to yield the final output, calculated as follows:

2 $$\left\{ \begin{gathered} \overset{\lower0.5em\hbox{$\smash{\scriptscriptstyle\rightharpoonup}$}} {h} _{t} = \sigma \left( {\omega _{1} x_{t} + \omega _{2} \overset{\lower0.5em\hbox{$\smash{\scriptscriptstyle\rightharpoonup}$}} {h} _{{t - 1}} } \right) \hfill \\ \overset{\lower0.5em\hbox{$\smash{\scriptscriptstyle\leftharpoonup}$}} {h} _{t} = \sigma \left( {\omega _{3} x_{t} + \omega _{4} \overset{\lower0.5em\hbox{$\smash{\scriptscriptstyle\leftharpoonup}$}} {h} _{{t + 1}} } \right) \hfill \\ y_{t} = g\left( {\omega _{5} \overset{\lower0.5em\hbox{$\smash{\scriptscriptstyle\rightharpoonup}$}} {h} _{t} + \omega _{6} \overset{\lower0.5em\hbox{$\smash{\scriptscriptstyle\leftharpoonup}$}} {h} _{t} } \right) \hfill \\ \end{gathered} \right.$$

where $$\overset{\lower0.5em\hbox{$\smash{\scriptscriptstyle\rightharpoonup}$}}{h} _{t}$$ and $$\overset{\lower0.5em\hbox{$\smash{\scriptscriptstyle\leftharpoonup}$}} {h} _{t}$$ indicated the outputs from both the forward and backward LSTM networks, respectively; $$y_{t}$$ represented the hidden layer’s final output, and $$\sigma ( \cdot )$$ and $$g( \cdot )$$ served as the respective activation functions.

## The hybrid bearing fault diagnosis model with HGWA optimization

### A hybrid one-dimensional CNN-BiLSTM network

**Fig. 3:**
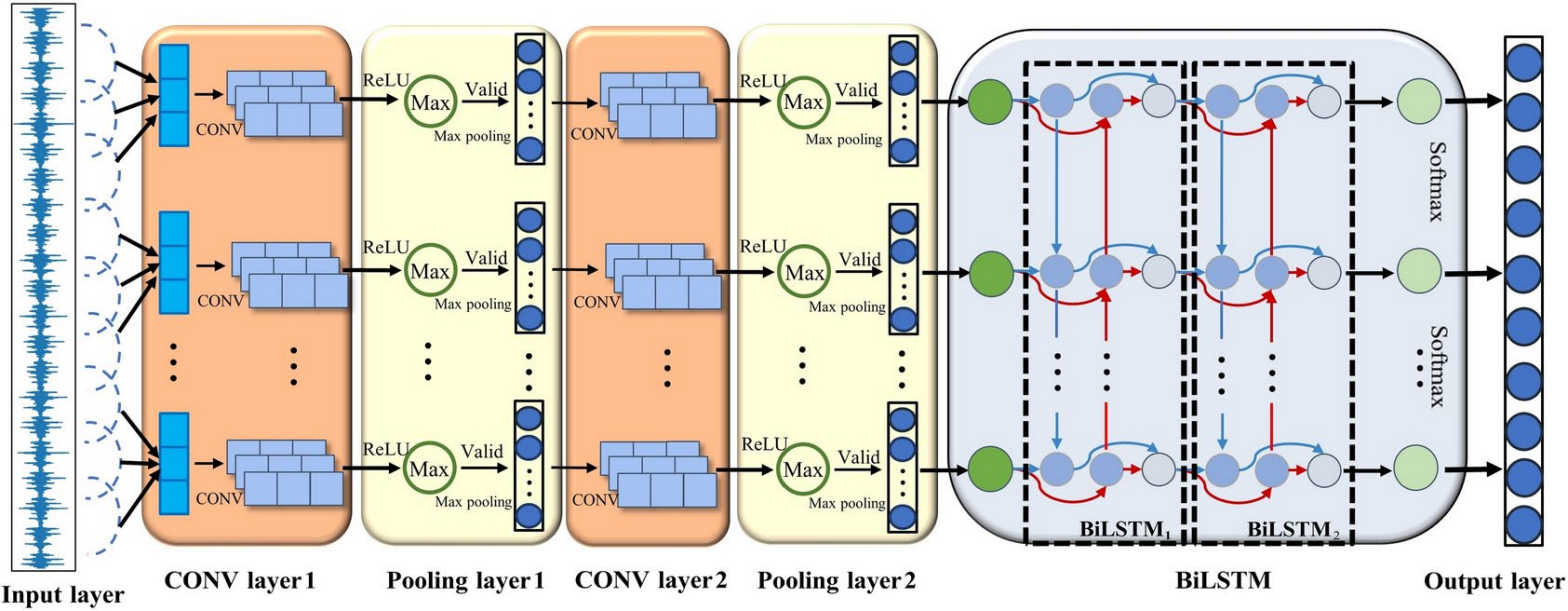
Network structure of the CNN-BiLSTM model.

In this study, a custom-designed one-dimensional CNN-BiLSTM network was developed for the feature extraction and classification of vibration signals. Generally, The CNN component is adept at extracting spatial features from the input data and reducing its dimensionality through convolutional kernels, while the BiLSTM network captures temporal correlations and bidirectional dependencies within the time-domain signals. By leveraging its memory cells and gate mechanisms, BiLSTM effectively analyzes both forward and backward data sequences. This study integrates the CNN network with the BiLSTM network, aiming to achieve comprehensive feature extraction from input signals. This combination not only compensates for the limitations of CNNs in time series analysis but also enhances the accuracy and efficiency of fault diagnosis. As illustrated in Fig. 3, The architecture of the CNN-BiLSTM model, includes an input layer, two convolutional layers, two pooling layers, two BiLSTM layers, a fully connected layer, and an output layer. This configuration enables effective processing of complex data, facilitating accurate and reliable fault identification. The basic diagnostic process follows these steps:

1. Collected vibration signals from the rolling bearing and segmented them into fixed intervals to create a dataset.
2. Input the dataset into the CNN convolutional layers, where convolutional kernels adaptively extracted fault features.
3. Applied max pooling on these extracted features within the pooling layers to reduce dimensionality while preserving essential feature information.
4. Fed the reduced-dimensional feature data into the BiLSTM layers, allowing the neural network to learn fault features automatically.
5. Utilized the Softmax activation function to classify the bearing fault features, thus completing the fault diagnosis.

### Hyperparameter optimization based on hybrid grey wolf algorithm

The GWO algorithm, which simulates the hunting behavior of wolves, is an effective intelligent optimization algorithm to solve complex optimization problems. The GWO algorithm is well-known for its high accuracy, stability, and good convergence, as well as its excellent generality and potential for further enhancements and extensions^46^. It strikes a balance between exploration and exploitation, and thus has been successfully applied in a number of domains, especially in the optimization of the Internet of Things (IoT). However, despite these advantages, the GWO algorithm still has certain limitations in its final stage. As a large number of grey wolf individuals approach the decision level, the diversity of the population decreases. This reduction in diversity may lead to premature convergence of the optimization, especially in the presence of locally optimal solutions at the decision layer. To make up for these shortcomings, this paper proposed a novel procedure that embeds the crossover and mutation operations of Genetic algorithm into the GWO algorithm. This combination could substantially enhance GWO’s capabilities of global search and diversity maintenance, preventing premature convergence and improving optimization performance.

#### GWO algorithm

In the GWO algorithm, wolves were categorized into four groups: *α, β, δ*, and *ω*. Throughout the optimization process, α represented the current optimal solution, *β* the second-best, and *δ* the third-best, while ω included the remaining candidate solutions. Consequently, the algorithm primarily depended on the three top categories: *α*, *β*, and *δ*. Based on the natural behavior of grey wolves, the hunting process was divided into three main phases: 1) encircling the prey, 2) pursuing the prey, and 3) attacking the prey.

In the encirclement phase, the wolves surrounded the prey, positioning it at the center of the pack. Each wolf maintained a certain distance from the prey. To simulate this behavior, the distance between a wolf and the prey, denoted as, $\overrightarrow {D}$ was calculated as shown in Eq. (3). Each wolf’s position was continuously updated based on the prey’s position, as illustrated in Eq. (4). Through this approach, GWO dynamically adjusted the wolves’ positions, thereby improving the algorithm’s search capability and convergence performance.

3 $$\vec{D} = \left| {\vec{C} \times \vec{X}_{P} \left( t \right) - \vec{X}\left( t \right)} \right|$$

4 $$\vec{X}\left( {t + 1} \right) = \vec{X}_{P} \left( t \right) - \vec{A} \times \vec{D}$$

where, *t* represents the current iteration number, *A* and *C* are coefficient vectors, denotes the position of the prey, and *X*(*t*) denotes the position of the wolf. The calculations for the coefficient vectors *A* and *C* are shown in Eqs. (5) and (6).

5 $$\vec{A} = 2\vec{a} \times \vec{r}_{1} - \vec{a}$$

6 $$\vec{C} = 2\vec{r}_{1}$$

where $\vec{r}_{1}$ and $\vec{r}_{2}$ were stochastic vectors in [0,1], and the calculation of the coefficient $\vec{a}$ was provided in Eq. (7). The coefficient *a* decreased from 2 to 0 as *t* increased, and $\vec{A}$ was determined by $\overrightarrow {a}$ When $\left| {\vec{A}} \right| < 1$, the search agent transitioned from the exploration phase to the exploitation phase.

7 $$\vec{a} = 2 - \frac{2 \times t}{T}$$

where *T* is the max number of iterations.

During the hunting stage, the positions of the wolves are updated according to Eq. (8).

8 $$\vec{X}\left( {t + 1} \right) = \frac{{\vec{X}_{1} + \vec{X}_{2} + \vec{X}_{3} }}{3}$$

where $\vec{X}_{1} ,\vec{X}_{2}$ and $\vec{X}_{3}$ are decided by the distance between $, ,$ and the prey respectively, shown in (9), (10), and (11).

9 $$\vec{X}_{1} = \vec{X}_{\alpha } - \vec{A}_{1} \times \vec{D}_{\alpha }$$

10 $$\vec{X}_{2} = \vec{X}_{\beta } - \vec{A}_{2} \times \vec{D}_{\beta }$$

11 $$\vec{X}_{3} = \vec{X}_{\delta } - \vec{A}_{3} \times \vec{D}_{\delta }$$

where $\vec{X}_{\alpha } {,}\;\vec{X}_{\beta } {,}\;\vec{X}_{\delta }$ are the position of *α*, $\beta$ and $\delta$ respectively. $\vec{D}_{\alpha } ,\vec{D}_{\beta }$ and $\vec{D}_{\delta }$ are the distance between *α*, $\beta$ ,$\delta$ and the prey respectively, shown in (12), (13), and (14).

12 $$\vec{D}_{\alpha } = \left| {\vec{C}_{1} \cdot \vec{X}_{\alpha } - \vec{X}_{1} } \right|$$

13 $$\vec{D}_{\beta } = \left| {\vec{C}_{2} \cdot \vec{X}_{\beta } - \vec{X}_{2} } \right|$$

14 $$\vec{D}_{\delta } = \left| {\vec{C}_{3} \cdot \vec{X}_{\delta } - \vec{X}_{3} } \right|$$

#### GA-GWO algorithm

The GWO algorithm simulates the grey wolf hunting process to optimize the objective function, but it may fall into local optima and lack sufficient global search capability, leading to decreased population diversity over time. To remedy these shortcomings of GWO algorithm, a combination of GA and GWO was originally developed in this section. GA is a search heuristic inspired by the process of natural selection, which mimics biological evolution. By improving population diversity and global search ability through selection, crossover, and mutation operations, GA can effectively compensate for the shortcomings of GWO. Specifically, the fast local search of GWO can make up for the shortcoming of the genetic algorithm’s slow convergence speed, while the global search and diversity maintenance mechanism of the GA can enhance the exploration ability of GWO. Introducing the crossover and mutation operations of the GA after each iteration of the GWO can essentially increase the population diversity and prevent premature convergence to the local optimum. With this complementary combination, the global search and local search can be better balanced to improve the overall optimization performance of the hybrid algorithm.

In the hybrid approach, the population is first initialized. Then, genetic operations, as described by Eqs. (15) and (16), are applied, including selection and mutation. The individual with the highest fitness is selected to form the new population. This process ensures that the most optimal solutions are retained and further refined in subsequent iterations, promoting the algorithm’s ability to resolve complex optimization problems with better efficiency and accuracy.

1. Crossover

New individuals are generated by exchanging some of the genes of two parent individuals. The crossover strategy formula is:

15 $$\left\{ {\begin{array}{*{20}l} {X_{c1}^{\prime } = \left[ {X_{1} \left[ {0{:}k_{1} } \right],\;X_{2} \left[ {k_{1} {:}k_{2} } \right],\;X_{1} \left[ {k_{2} {:}N} \right]} \right]} \hfill \\ {X_{c2}^{\prime } = \left[ {X_{2} \left[ {0{:}k_{1} } \right],\;X_{1} \left[ {k_{1} {:}k_{2} } \right],\;X_{2} \left[ {k_{2} {:}N} \right]} \right]} \hfill \\ \end{array} } \right.$$

where $k_{1}$ and $k_{2}$ is the crossover point and *N* is the length of the individual. $X_{1} ,X_{2}$ are the parents and $X_{c1}^{\prime } ,X_{c2}^{\prime }$ are the children.

(2) Mutation

Randomly change some genes in new individuals to introduce diversity. The mutation strategy formula is:

16 $$\vec{X}_{m}^{\prime } {[}j{]} = \left\{ {\begin{array}{*{20}l} {{\text{Random(bounds[}}j{])}} \hfill & {{\text{if}}\;{\text{rand()}} < {\text{mutation}}\;{\text{rate}}} \hfill \\ {\vec{X}{[}j{]}} \hfill & {{\text{otherwise}}} \hfill \\ \end{array} } \right.$$

where ${\text{Random(bounds[}}j{])}$ denotes the generation of a new value randomly within the range of values of the $j$ gene.

(3) Arithmetic crossover operation

For three individuals $\vec{X}_{r1} ,\vec{X}_{r2}$ and $\vec{X}_{r3}$ randomly selected from the population, $\vec{X}_{\alpha } {, }\vec{X}_{\beta }$ and $\vec{X}_{\delta }$ individuals and in the decision hierarchy, the new position update formula is:

17 $$\vec{X}_{{{\text{new}}}} = \vec{X}_{\alpha } + a \cdot {(}\vec{X}_{r1} - \vec{X}_{r2} {)} + \theta \cdot {(}\vec{X}_{r3} - \vec{X}_{\alpha } {)}$$

where $\vec{X}_{\alpha } {, }\vec{X}_{\beta } {, }\vec{X}_{\delta }$ are the position of *α*, $\beta$ and $\delta$ respectively. $a$,$\theta$ are random numbers between (0, 1) and are updated at each iteration.

(4) Cross-operation

In order to further improve the local search capability, the following crossover operation is performed on the formulas of $\vec{X}_{{{\text{new}}}}$, $\vec{X}_{\beta }$, and $\vec{X}_{\delta }$ with reference to Eq. (4):

18 $$\left\{ \begin{gathered} \vec{X}_{\beta new}^{c} = {\text{Crossover(}}\vec{X}_{{{\text{new}}}} {,}\;\vec{X}_{\beta } {)} \hfill \\ \vec{X}_{\delta new}^{c} = {\text{Crossover(}}\vec{X}_{{{\text{new}}}} {,}\;\vec{X}_{\delta } {)} \hfill \\ \end{gathered} \right.$$

(5) Mutation operation

The mutation operation introduces randomness to increase the diversity of the population. The variation formula is:

19 $$\vec{X}_{new}^{m} {[}j{]} = \left\{ {\begin{array}{*{20}l} {{\text{Random(bounds[}}j{])}} \hfill & {{\text{if}}\;{\text{rand()}} < {\text{mutation}}\;{\text{rate}}} \hfill \\ {\vec{X}_{new} {[}j{]}} \hfill & {{\text{otherwise}}} \hfill \\ \end{array} } \right.$$

where rand ( ) is a random number in the range [0, 1].

(6) Update the positions of *α*, *β* and *δ*

The three individuals with the highest fitness were selected as the new *α, β* and* δ* individuals from the current population and the newly generated individuals:

20 $${\text{\{ }}\vec{X}_{\alpha }^{{_{\prime } }} {,}\vec{X}_{\beta }^{{_{\prime } }} {,}\vec{X}_{\delta }^{{_{\prime } }} {\text{\} }} = TOP3\left\{ {\vec{X}_{\left( i \right)} {,}\vec{X}_{\left( i \right)\;new} } \right\}$$

where $\vec{X}_{\left( i \right)}$ is the location of all agents in the current population $\vec{X}_{\left( i \right)\;new}$ is the location of all agents in the nascent population, and the three most adapted agents are retained as the new generation of leaders. The final position update formula is:

21 $$\vec{X}_{i} (t + 1) = \frac{{\vec{X}_{\alpha }^{\prime } (t + 1) + \vec{X}_{\beta }^{\prime } (t + 1) + \vec{X}_{\delta }^{\prime } (t + 1)}}{3}$$

##### Remark 1

The cross-entropy loss function is utilized as a criterion for evaluating the degree of agent adaptation, represented by the following equation:

22 $$F{(}a{)} = {(}L_{val} {(}a{)},A_{val} {(}a{))}\;\;$$

23 $$L_{val} (a) = - \frac{1}{{N_{val} }}\sum \frac{{N_{val} }}{i = 1}\sum \frac{C}{c = 1}y_{ic} log(\hat{y}_{ic} )$$

24 $$A_{val} {(}a{)} = \frac{1}{{N_{val} }}\;\sum \frac{{N_{val} }}{i = 1}\;1\left( {arg\;max_{c} \hat{y}_{ ic} = arg\;max_{c} y_{ic} } \right)$$

25 $$a = \left\{ {kernel\;size,\;filters,\;BiLSTM\;units,\;learningrate} \right\}$$

where $L_{val}$ is denotes the loss of the model *a* on the validation set;$A_{val} {(}a{)}$ is denotes the model *a* accuracy on the validation set; *N* indicates the number of samples in the batch; *C* indicates the number of categories; $y_{ic}$ denotes the first one-hot coded value of the true category of the *i*th sample; $\hat{y}_{ic}$ denotes the first *i*th sample is predicted to be the category *c* with probability.

### Bearing fault diagnosis based on hybrid CNN-BiLSTM model with HGWA optimization

**Fig. 4:**
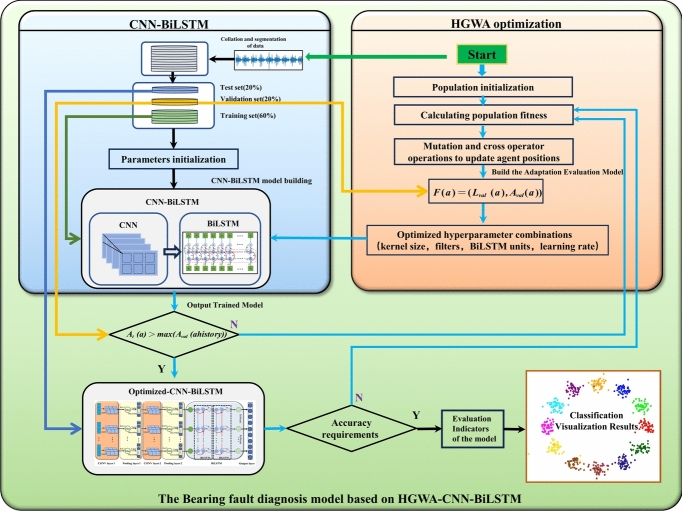
The HGWA-Optimized Hybrid CNN-BiLSTM Network bearing fault diagnosis model structure.

In CNN and BiLSTM networks, hyperparameter selection has had a notable impact on model performance, and optimization algorithms have increasingly been used to fine-tune these hyperparameters. Previous studies have mainly focused on optimizing aspects such as kernel size, filter quantity, and network depth to enhance performance. However, training parameters have also proven critical for modeling, as they directly influence the accuracy and reliability of the trained model. For example, the size of the convolutional kernel has determined the effectiveness of feature extraction; using too many filters has increased computational complexity, while too few filters have resulted in inadequate feature capture. A high learning rate has hindered model convergence, whereas a low learning rate has slowed it down. Insufficient iterations have led to underfitting, while too many iterations have caused overfitting. The batch size has needed careful balancing to maintain both training efficiency and stability. Large strides have overly compressed the feature map, while small strides have increased computational cost. Proper padding has preserved edge information and improved feature extraction. Based on these considerations, the convolutional kernel size, the number of filters, and the number of neurons in the hidden layer of BiLSTM were chosen to be the optimization objectives in this study. Optimizing these key parameters has significantly enhanced the model’s overall performance, along with its training and learning processes, making the final model more accurate and robust in practical applications. Figure 4 presents a flowchart of the CNN-BiLSTM model optimized with HGWA for bearing fault diagnosis, which is described in the following manner:

*Step 1* Normalize and oversample raw vibration signals to generate sequences for model training and testing.

*Step 2* Label and shuffle the sequence samples, then split them into training, validation, and test sets according to predefined ratios.

*Step 3* Initialize the HGWA algorithm to create hyperparameter particles for the CNN-BiLSTM model, which include parameters such as learning rate, kernel size, filter count, and BiLSTM hidden units.

*Step 4* The CNN-BiLSTM model is initialized with the specified hyperparameters, and the samples are provided for training, validation, and testing. The validation cross-entropy loss is then used to evaluate the fitness score for the HGWA optimization process.

*Step 5* The positions of the hyperparameter particles in the HGWA search space are updated according to their respective fitness values.

*Step 6* Check if the stopping criteria are met; if met, output the optimization results. Otherwise, return to Step 4 and repeat the process.

## Diagnosis of bearing faults across various operating conditions

In practical engineering applications, the feature distribution of the collected signals from different fault scenarios is often inconsistent due to varying operating conditions, different physical properties, ambient noise, and different sensors^47–50^. Additionally, it is quite difficult for mechanical devices to collect monitoring data for extended periods under faulty or near-fault conditions, resulting in scarce or even non-existent fault data. To tackle these challenges, various Deep Transfer Learning (DTL) methods were developed, being capable of transferring knowledge from well-labeled samples in the source domain to related but different unlabeled target domains^51,52^. DTL methods have generally been classified into instance-based, model-based, feature-based, and relationship-based approaches^53^. I In the field of Preventive Health Management (PHM), model-based strategies have become especially common, as they leverage models trained in one domain to support another, related domain. Typical applications have included direct use of pre-trained models, fine-tuning, and model stacking^54^, each adapting differently according to model specifications.

**Fig. 5:**
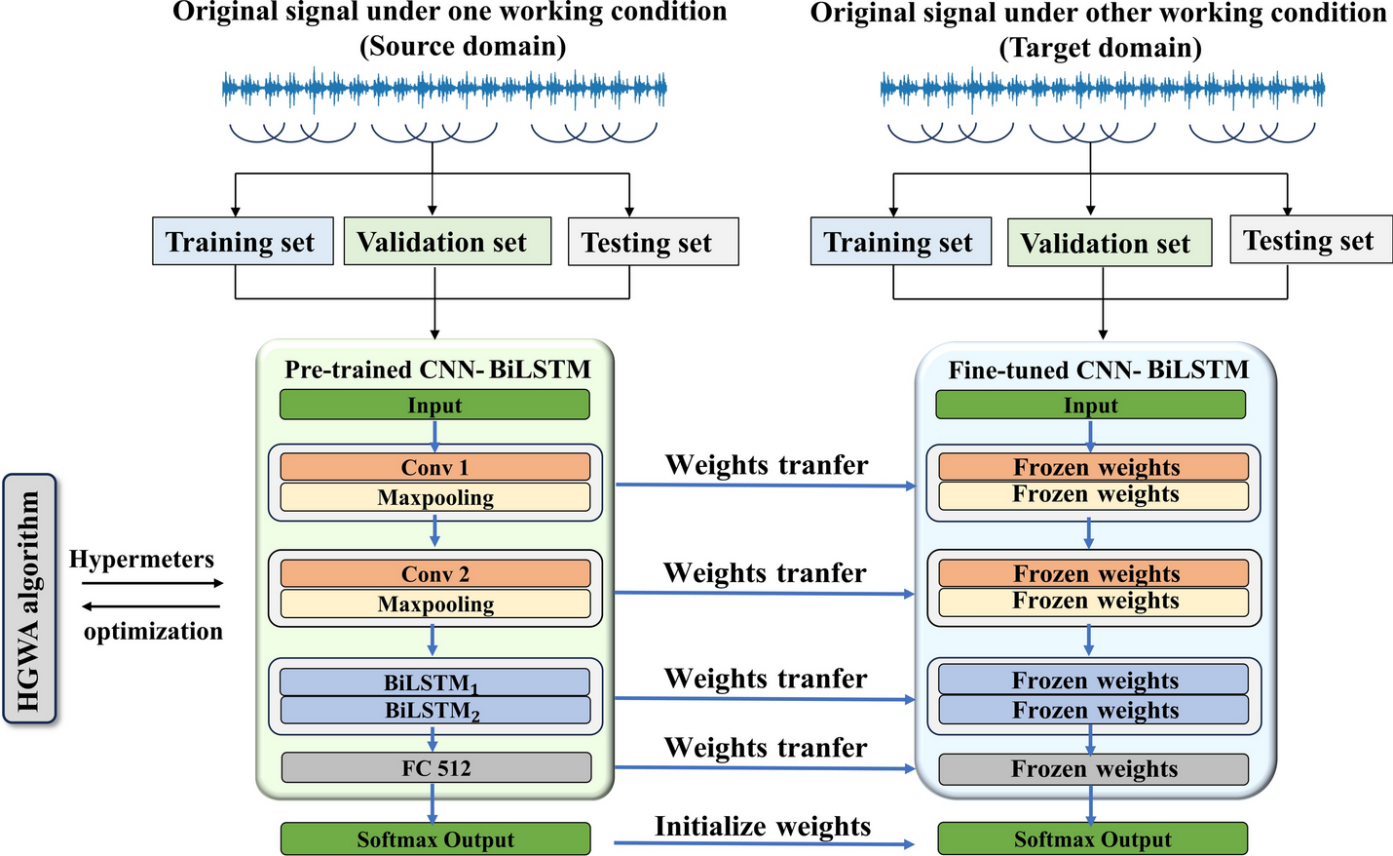
Framework for bearing fault diagnosis under multiple operating conditions.

Utilizing pre-trained models to initialize the weights of target models, the model-based transfer learning approach can be employed to carry out fault diagnosis under a wide range of operating conditions. In this study, the incorporation of the hybrid CNN-BiLSTM network and HWGA optimization algorithm could substantially improve the diagnostic performance and the generalization ability of the trained model, achieving high accuracy even with limited sample sizes. Accordingly, the application of a model-based transfer learning approach can achieve efficient and accurate diagnosis with minimal samples from the target domain, effectively diminishing the difficulty of collecting sufficient training data under different operating conditions of rolling bearings. A fault diagnosis framework for multi-operating conditions has been established, as shown in Fig. 5. The pre-trained model, a 9-layer network, has been optimized with the HGWA algorithm for accurate fault classification using a dataset from a specific operating condition. In contrast, the target domain dataset, collected under different conditions, includes a small subset for training. The weights of 7 intermediate layers (excluding input and output layers) from the pre-trained model have been transferred to the target model, with some weights frozen and others fine-tuned to enhance diagnostic accuracy despite limited data. During fine-tuning, only the output layer weights have been retrained with the target training set. A separate test dataset has then been used to validate the model, enabling precise classification of various fault types under these conditions. The multi-condition bearing fault diagnosis process comprises the following steps:

*Step 1* Vibration signals from bearings under various conditions have been collected and processed to create input datasets.

*Step 2* The data has been divided into training, validation, and test sets for model training, evaluation, and final performance assessment.

*Step 3* A CNN-BiLSTM network has been pre-trained on a specific dataset, optimized with the HGWA algorithm to establish a source domain model for transfer learning.

*Step 4* The model has been fine-tuned with a subset from a different condition, freezing certain layers while updating others to enhance performance.

*Step 5* The fine-tuned model has been saved and validated using a test dataset to ensure accuracy in fault diagnosis.

### Remark 2

The CNN-BiLSTM model learns features from the input data in an autonomous manner. The lower layers focus on extracting fundamental features such as edges and curves, while the higher layers are responsible for identifying more intricate, domain-specific patterns. Model-based transfer learning (TL) leverages this capability by retaining lower-layer weights and focusing on new, high-level feature learning with fresh data. TL improves training efficiency significantly by requiring fewer parameters to be retrained, in contrast to training a model from scratch.

### Remark 3

If the model from the source domain demonstrates strong generalization capabilities, fine-tuning with a small amount of target domain data can still achieve high performance. When significant differences exist between the source and target domain data, or when the source model lacks adequate stability and generalization, it becomes essential to modify additional layers and weight parameters during the fine-tuning phase.

## Experimental results

### Experimental datasets and model parameters

#### Experimental datasets

**Fig. 6:**
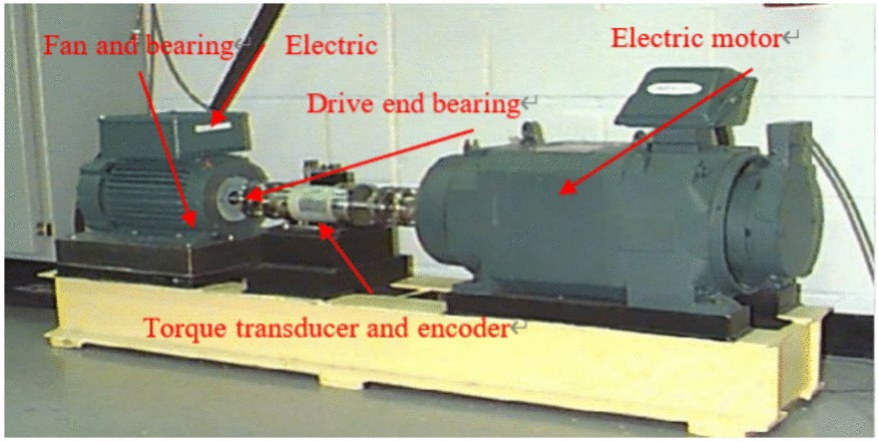
Experimental system for CWRU dataset.

In this study, fault data from rolling bearings was obtained from two widely recognized datasets: the CWRU dataset^49^ and the JNU dataset, both of which are crucial for research in bearing fault diagnosis. The experimental setup for the CWRU dataset is illustrated in Fig. 6, where a 1.5 kW asynchronous motor powers a fan, which is connected to a power meter and torque sensor through a self-calibrating coupler. Vibration acceleration data was collected during fault diagnosis experiments using an SKF6205 deep groove ball bearing, manufactured by SKF, as a representative sample. In contrast, the JNU dataset was recorded under similar experimental conditions using a 2.2 kW motor, encompassing 12 distinct bearing conditions, thus providing a comprehensive basis for fault diagnosis analysis.

#### Experimental model parameters

The experimental setup for this study includes an Intel Core i5-12490KF processor operating at 3.00 GHz, paired with 32 GB of RAM. PyCharm was utilized as the development environment, and Keras 2.3.1 served as the deep learning framework.

As detailed in Table 1, the one-dimensional CNN-BiLSTM model designed for bearing fault classification includes an input layer with a sample size of 1024, followed by two convolutional layers and two pooling layers. The kernel size and number of filters in the convolutional layers have been optimized, while the pooling layer utilizes a 2 × 1 kernel with stride settings of 1 × 1 for convolution and 2 × 1 for pooling. The BiLSTM layer’s neuron count has also been optimized, and the fully connected layer comprises 512 neurons. A softmax function is applied in the output layer to classify vibration signals into 10 distinct categories, each representing a different fault type. Convolution operations use “same” padding, and “max pooling” is applied in the pooling layers. The ReLU activation function is employed in both the convolutional and fully connected layers.

**Table 1:** Architecture of the introduced 1-D CNN-BiLSTM Network.

Structure	No.	Layers	Kernel size/Stride	Output	Parameters
Input	1	Input	–	(None, 1024, 1)	0
CNN	2	Conv 1	Kernel_size/1	(None, 1024, n_filters)	Varies
	3	Pooling 1	2*1/2*1	(None, 512, n_filters)	0
	4	Conv 2	Kernel_size/1	(None, 512, n_filters)	Varies
	5	Pooling 2	2*1/2*1	(None, 256, n_filters)	0
BiLSTM	6	BiLSTM 1	–	(None, 256, 2 * n_units)	Varies
	7	BiLSTM 2	–	(None, 256)	Varies
Fully-connected	8	Fully-connected	–	(None, 512)	Varies
Output	9	Softmax	–	(None, 10)	Varies

Since the initial learning rate, convolutional kernel size, the number of filters, and the number of neurons are used as the optimization targets, their values will change within a certain range according to the optimization process. The optimization ranges for these four important hyperparameters are presented in Table 2.

**Table 2:** Interval setting of the CNN-BiLSTM hyperparameters.

Hyperparameters	Interval
Kernel size	[3,32]
Filters	[3,128]
BiLSTM units	[3,128]
Learning rate	[10^–4^,10^–2^]

### Experimentation on the CWRU Dataset

The CWRU dataset includes four different types of bearing fault conditions: no fault (NF), ball fault (BF), inner-ring fault (IF), and outer-ring fault (OF). These faults were induced on the bearing surfaces using discharge machines, with fault diameters of 0.007 inches, 0.014 inches, and 0.021 inches. Vibration data were collected through an accelerometer attached to the motor drive, with a sampling frequency set at 12 kHz. As detailed in Table 3, the motor was tested under different load conditions, including 0 hp, 1 hp, 2 hp, and 3 hp. From these various test conditions, four separate bearing fault datasets were created and labeled A, B, C, and D. Each dataset represents 10 fault categories, including one for no fault, and covers different damage levels and fault locations on the bearing. This resulted in a fault classification system with 10 labels, ranging from 0 to 9. To enhance the dataset and improve classification accuracy, the vibration signals were sampled using an overlapping method, where each sequence segment contained 1024 data points and had a step size of 150. A total of 250 samples were gathered for each fault type, resulting in 2500 samples per dataset.

**Table 3:** Description of bearing fault types.

Fault	Name	Diameter (inch)	Load (hp)/Dataset	Label
NF	NF000	0	0/A, 1/B, 2/C, 3/D	0
BF	BF007	0.007	0/A, 1/B, 2/C, 3/D	1
	BF014	0.014	0/A, 1/B, 2/C, 3/D	2
	BF021	0.021	0/A, 1/B, 2/C, 3/D	3
	IF007	0.007	0/A, 1/B, 2/C, 3/D	4
IF	IF014	0.014	0/A, 1/B, 2/C, 3/D	5
	IF021	0.021	0/A, 1/B, 2/C, 3/D	6
	OF007	0.007	0/A, 1/B, 2/C, 3/D	7
OF	OF014	0.014	0/A, 1/B, 2/C, 3/D	8
	OF021	0.021	0/A, 1/B, 2/C, 3/D	9

#### Fault diagnosis based on the hybird CNN-BiLSTM model

Each dataset, which corresponds to various operating conditions, consists of 250 samples for each type of bearing fault. These samples were split into training, validation, and testing sets using a 6:2:2 ratio, resulting in 150 samples for training, 50 for validation, and 50 for testing per fault type. To assess the performance of the CNN-BiLSTM hybrid model for fault diagnosis, we conducted comparative experiments with two alternative models, CNN and CNN-LSTM, all of which had identical configurations for their hidden layers. The experiments were executed across datasets A, B, C, and D. For training, all models employed a cross-entropy loss function, the Adam optimizer with a learning rate of 0.004, a batch size of 32, and a total of 10 epochs. To minimize randomness, each experiment was repeated 10 times, and the average diagnostic accuracy was computed.

This study utilized accuracy, precision, recall, and macro F1-score as evaluation metrics, with detailed results presented in Table 4. Analysis of accuracy within each dataset revealed consistent improvements in fault classification performance. Among the models tested, the CNN-BiLSTM achieved the highest average classification accuracy across all four datasets, demonstrating its superior learning and generalization capabilities in comparison to the LSTM network. Specifically, the three models achieved average accuracies of 98.07%, 98.55%, and 99.23%, respectively, indicating robust performance under various conditions. Moreover, CNN-BiLSTM and CNN-LSTM outperformed the CNN model by 1.16% and 0.48%, respectively, further highlighting BiLSTM’s enhanced feature extraction capabilities over LSTM.

**Table 4:** Comparison on four fault diagnosis models.

Datasets	Model	Accuracy (%)	Precision (%)	Recall (%)	F1-score (%)
A	CNN	97.892	97.945	97.892	97.875
	CNN-LSTM	98.360	98.492	98.360	98.337
	CNN-Transformer	98.645	98.637	98.659	98.642
	CNN-BiLSTM	99.135	99.271	99.135	99.112
B	CNN	97.785	97.800	97.785	97.780
	CNN-LSTM	98.273	98.306	98.273	98.268
	CNN-Transformer	98.945	98.952	98.946	98.950
	CNN-BiLSTM	99.025	99.062	99.025	99.021
C	CNN	98.214	98.34	98.214	98.192
	CNN-LSTM	98.649	98.732	98.649	98.627
	CNN-Transformer	99.148	99.150	99.151	99.146
	CNN-BiLSTM	99.347	99.479	99.347	99.324
D	CNN	98.397	98.602	98.397	98.383
	CNN-LSTM	98.899	98.937	98.899	98.877
	CNN-Transformer	99.236	99.232	99.229	99.238
	CNN-BiLSTM	99.402	99.428	99.402	99.398

**Fig. 7:**
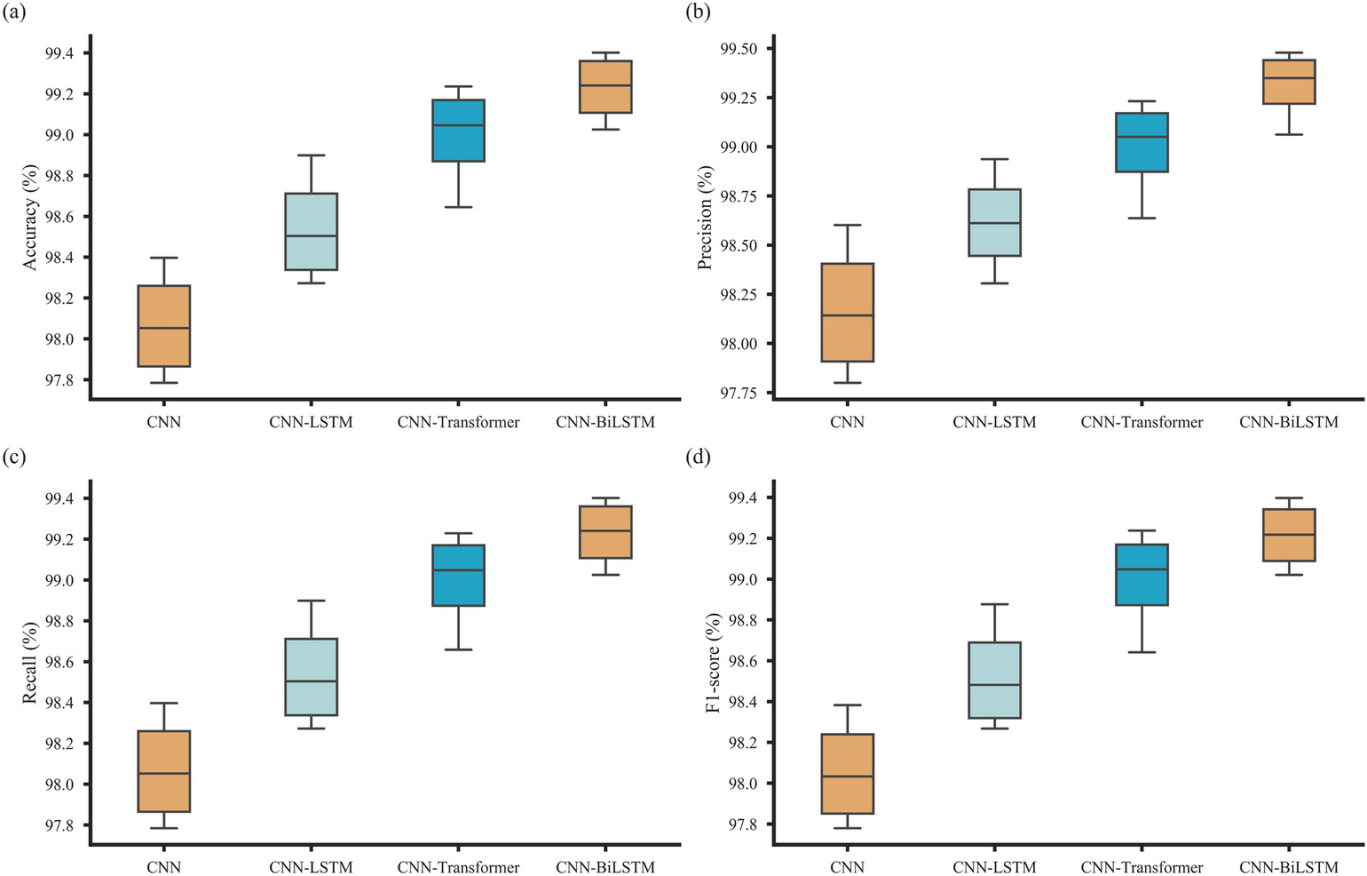
Box plots of the experimental results: (**a**) Accuracy, (**b**) Precision, (**c**) Recall, (**d**) F1-score.

Figure 7 presents box plots that depict the experimental results under four distinct conditions. Among the models evaluated, the CNN consistently demonstrated the lowest accuracy on the test set, with the CNN-LSTM achieving slightly improved performance. In contrast, the CNN-BiLSTM model surpassed all others, achieving the highest accuracy and demonstrating superior predictive stability. As model complexity increased, a clear trend of enhanced prediction stability emerged, highlighting the advantages of employing more sophisticated architectures to improve performance.

The inclusion of the CNN-Transformer model provides additional insights into the potential of advanced architectures. While the CNN-Transformer exhibited notable improvements over CNN and CNN-LSTM in both accuracy and stability, it did not outperform the CNN-BiLSTM in this experimental setting. This outcome likely reflects the CNN-BiLSTM’s unique ability to better capture intricate data features and fine-grained temporal dependencies, which are critical for achieving optimal performance in this context. Nevertheless, the CNN-Transformer demonstrates strong temporal correlation capabilities, marking it as a competitive and promising alternative.

These findings underscore the efficacy of the CNN-BiLSTM model in accurately representing complex data and enhancing temporal correlations. While CNN-Transformer offers a compelling architecture with considerable advantages, the results reaffirm the CNN-BiLSTM’s superiority in achieving both accuracy and stability across diverse experimental conditions.

##### Remark 4

In addition to accuracy, other evaluation metrics such as precision, recall, and macro F1-score further reinforce the previous analysis, providing a comprehensive evaluation of model performance across varying operating conditions. The CNN-BiLSTM model consistently outperformed not only the CNN and CNN-LSTM models but also the CNN-Transformer in these metrics, highlighting its exceptional capability to extract features and capture temporal dependencies effectively. While the CNN-Transformer demonstrated improvements over CNN and CNN-LSTM in precision and recall, it still fell short of the CNN-BiLSTM’s superior performance, underscoring the latter’s robustness and efficiency in handling complex temporal data.

#### Fault diagnosis using the hybird CNN-BiLSTM model integrated with HGWA optimization

This section introduces the application of the enhanced HGWA variant of the GWO algorithm to optimize the CNN-BiLSTM model’s hyperparameters for effective bearing fault diagnosis. This method enhances diagnostic accuracy and efficiency by automating parameter selection, thus reducing reliance on manual tuning. Using a particle-based representation, the HGWA algorithm searches the defined parameter space to identify the optimal hyperparameter set that minimizes cross-entropy loss, as shown in Table 2.

In order to rigorously assess the performance of the HGWA algorithm, five well-established optimization algorithms—PSO with adaptive weighted delay velocity (PSO-AWDV), Hybrid Particle Swarm Optimization (HPSO), Bayesian Optimization (BO), Random Search (RS), and Grid Search (GS)—were incorporated into the hybrid CNN-BiLSTM model to compare their efficacy in bearing fault diagnosis. The population size for the HGWA, PSO-AWDV, and HPSO algorithms was set to 200, while the mutation rate for HGWA was fixed at 0.1. For the RS and GS algorithms, cross-validation was set to 3. Additionally, the maximum number of iterations for all five optimization algorithms (HGWA, PSO-AWDV, HPSO, BO, GS, and RS) was set to 10.

Table 5 summarizes the mean performance metrics derived from 10 independent experimental trials. The HGWA-optimized CNN-BiLSTM architecture achieves perfect classification accuracy (100%) across all four evaluated datasets (A–D), marking a statistically significant advancement over the benchmarks reported in Table 4. Crucially, hybrid CNN-BiLSTM models integrated with four alternative optimization algorithms (RS, GS, HPSO, PSO-AWDV) underperform relative to HGWA under identical experimental conditions.

**Table 5:** Comparison on optimized CNN-BiLSTM fault diagnosis models.

Datasets	Model	Accuracy (%)	Precision (%)	Recall (%)	F1-score (%)	Time (s)
A	GS	98.232	98.257	98.232	98.169	5285
	RS	98.476	98.483	98.476	98.429	4686
	BO	99.686	99.682	99.686	99.662	3987
	HPSO	99.862	99.868	99.862	99.849	2494
	PSO-AWDV	99.925	99.926	99.925	99.925	2569
	HGWA	99.968	99.976	99.968	99.968	2596
B	GS	98.025	98.062	98.025	98.021	4908
	RS	98.950	98.936	99.039	99.036	4493
	BO	99.168	99.159	99.168	99.166	3621
	HPSO	99.406	99.417	99.406	99.489	2249
	PSO-AWDV	99.580	99.583	99.583	99.583	1605
	HGWA	99.860	99.879	99.860	99.858	1329
C	GS	99.374	99.390	99.374	99.358	5845
	RS	99.052	99.063	99.052	99.032	4819
	BO	99.360	99.356	99.360	99.358	4652
	HPSO	99.649	99.682	99.649	99.629	1708
	PSO-AWDV	99.790	99.794	99.790	99.799	2614
	HGWA	100	100	100	100	1087
D	GS	99.502	99.528	99.502	99.498	5708
	RS	98.899	98.917	98.899	98.873	4096
	BO	99.686	99.664	99.686	99.672	3809
	HPSO	99.899	99.917	98.899	98.874	2289
	PSO-AWDV	99.945	99.946	99.945	99.945	1902
	HGWA	99.978	99.602	99.978	99.966	968

Beyond exceptional accuracy, the HGWA-optimized framework demonstrates marked reductions in computational resource demands. By systematically refining training hyperparameters, HGWA yields a leaner network architecture, characterized by fewer trainable parameters, diminished memory utilization, and accelerated convergence rates. Comparative analyses reveal that HGWA surpasses RS, GS, HPSO, and BO in convergence speed, matching the efficiency of PSO-AWDV while maintaining superior accuracy. When constrained to equivalent training durations, the HGWA-optimized model consistently attains peak accuracy with minimal resource expenditure.

**Fig. 8:**
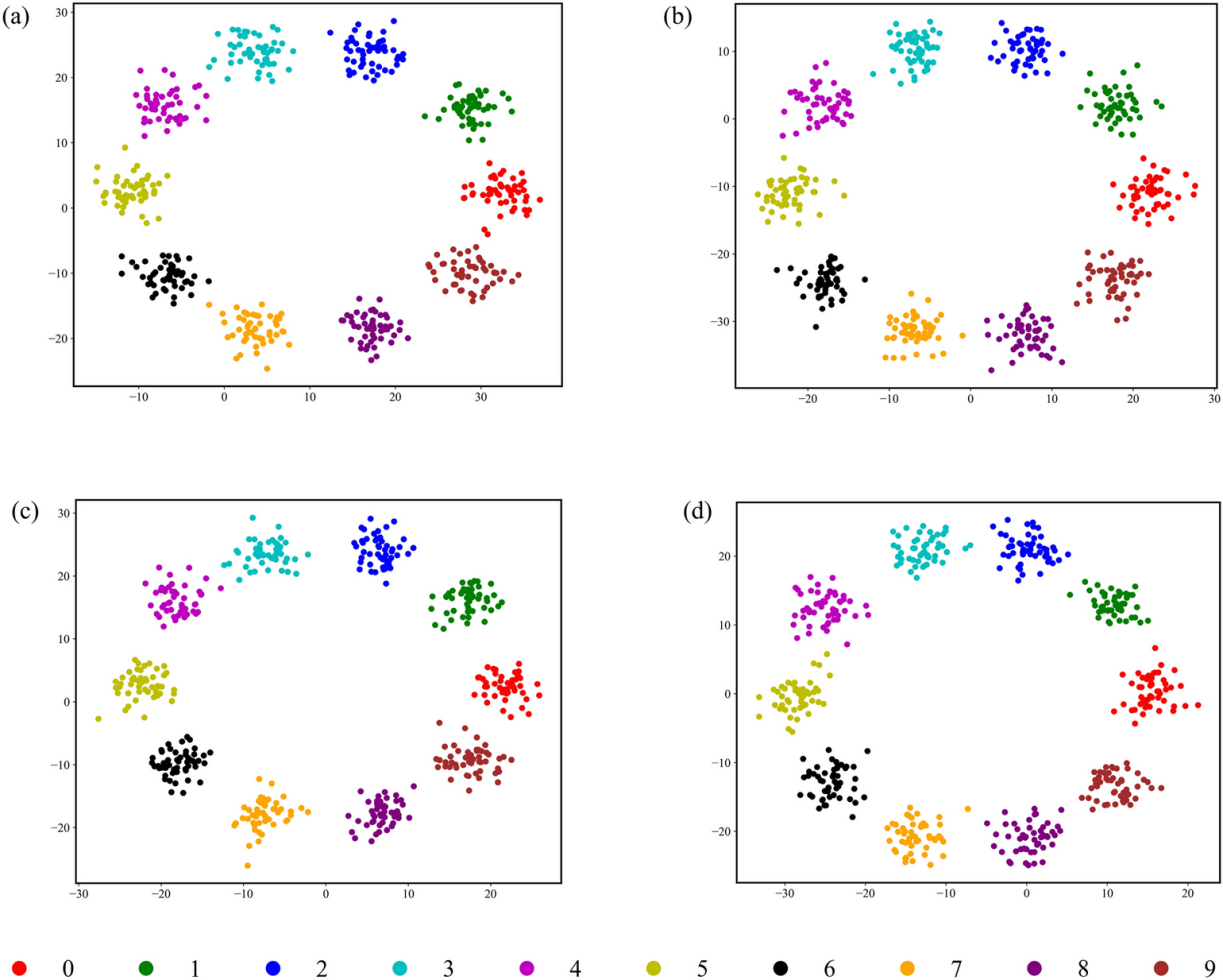
t-SNE visualization clustering results: (**a**) Datasets A, (**b**) Datasets B, (**c**) Datasets C, (**d**) Datasets D.

The efficacy of HGWA in balancing hyperparameter optimization with computational frugality enables the CNN-BiLSTM framework to achieve both state-of-the-art diagnostic precision and robust generalization. The algorithm’s capacity to minimize training overhead while preserving performance underscores its suitability for real-time industrial applications, where operational efficiency and diagnostic reliability are paramount. This dual optimization of accuracy and resource efficiency positions HGWA as a compelling solution for scalable, high-fidelity fault diagnosis in complex industrial systems. To ensure the robustness of the results presented in Table 6, we performed tenfold cross-validation on each dataset. The cross-validation results are summarized in Table 7, which shows the mean accuracy, standard deviation, and range of accuracy across all folds. The consistently perfect accuracy (100% for all datasets, e.g., Dataset A and Dataset B) with zero standard deviation (0.0%) confirms the model’s stability and generalization ability. Furthermore, Fig. 8 displays t-SNE-based visual clustering of the output features from the test set, illustrating the model’s exceptional performance with well-defined and distinguishable classification boundaries.

**Table 6:** Optimal results of HGWA optimized CNN-BiLSTM model.

Datasets	Accuracy (%)	Precision (%)	Recall (%)	F1-score (%)	Time (s)	Hyperparameter range [Kernel size, Filters, BiLSTM units, Learning rate]	Params
A	100	100	100	100	2843	[8, 124, 109, 0.001593]	4.42 M
B	100	100	100	100	1567	[15, 111, 38, 0.001215]	1.73 M
C	100	100	100	100	1087	[19, 61, 98, 0.000438]	1.52 M
D	100	100	100	100	1478	[24, 126, 43, 0.002452]	2.64 M

**Table 7:** Results of tenfold cross validation under four operating conditions.

Datasets	Mean accuracy (%)	Standard deviation (%)	Accuracy range (%)
A	100	0.0	100–100
B	100	0.0	100–100
C	100	0.0	100–100
D	100	0.0	100–100

#### Fault diagnosis across varying operational conditions

The varying and complex operating conditions of rolling bearings pose significant challenges in acquiring enough training data for accurate fault diagnosis across different scenarios. This section investigates how model-based transfer learning techniques can be utilized to tackle bearing fault diagnosis, particularly in situations where limited training data is available.

In these experiments, data from one operating condition of the CWRU dataset was used as the source domain, and data from the other three conditions were assigned as the target domains. To evaluate the effectiveness of the cross-condition fault diagnosis approach, experiments were performed with different amounts of training samples (10, 20, 30, and 40) from the target domains, while 500 samples were set aside for testing. The performance of the model was assessed under these varying conditions to determine its adaptability and robustness. The HGWA-optimized CNN-BiLSTM model, with frozen weights in all layers except the input and output layers, was fine-tuned using the training data. Training parameters included 10 iterations, a batch size of 32, a learning rate from the optimized pre-trained model, and the Adam optimizer. Each experiment was repeated 10 times to minimize variability, and the results were averaged.

Table 8 presents the classification accuracy results achieved through the application of model-based transfer learning. In the cross-condition fault diagnosis experiment, dataset A was employed as the source domain, while dataset D was designated as the target domain. With only 10 training samples, the model achieved an average accuracy of 86.8%, demonstrating the model’s performance under these conditions. This relatively low accuracy is largely attributed to the differing data characteristics between domains: the source domain corresponds to a no-load condition, whereas the target domain includes loaded conditions. The limited training data further contributed to this lower accuracy. However, as the quantity of training samples grew from 10 to 40, diagnostic accuracy saw a significant improvement, reaching 99.76%. This trend was consistently observed across other experiments. Across these 12 experiments, the average accuracy rose from 95.26 to 99.65% as sample size grew. These findings confirm that model-based transfer learning enables high classification accuracy even with limited sample sizes.

**Table 8:** Classification accuracy using model-based transfer learning.

Source	Target	Samples				
		0	10	20	30	40
A	B	–	93.4	98.88	99.4	99.68
	C	–	95.32	98.92	99.32	99.8
	D	–	86.8	98.48	99.68	99.76
B	A	16	94.6	98.48	99.4	99.6
	C	99.24	100	100	100	100
	D	90.88	96.92	99.04	99.16	99.76
C	A	10.02	91.4	97.4	98.38	99.02
	B	99.8	99.5	99.6	99.8	100
	D	99.4	93.6	99.85	99.92	100
D	A	13.8	96.8	97.8	97.96	98.6
	B	97.53	96.2	98.2	99.26	99.6
	C	98.88	98.6	99.2	99.8	100
Mean			95.26	98.82	99.34	99.65

**Fig. 9:**
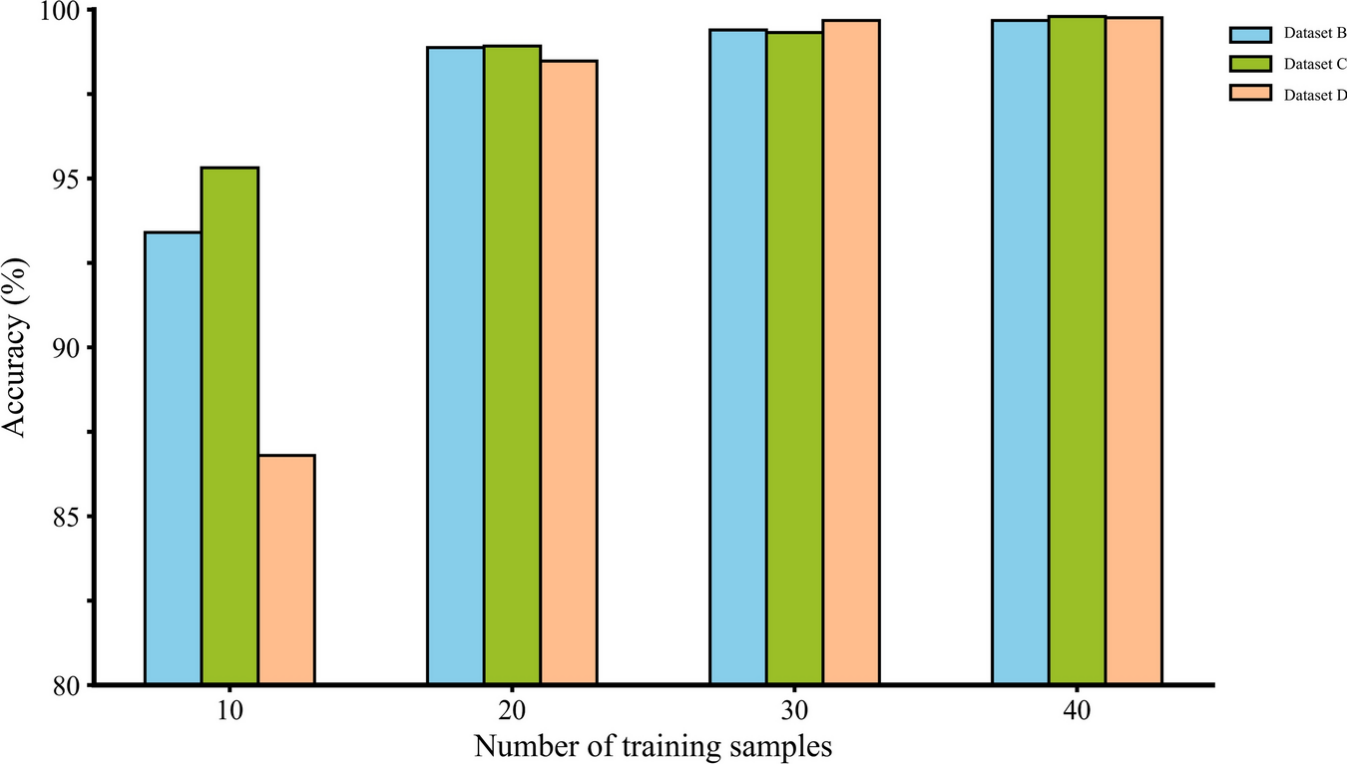
Classification accuracy across datasets B, C, and D using different training sample sizes from dataset A.

The bar chart in Fig. 9 clearly illustrates the experimental outcomes, showing that classification accuracy for the target domain model increases with a higher number of training samples. A notable improvement in accuracy is observed as the sample count rises from 10 to 20. These results suggest that transfer learning, combined with fine-tuning using limited data, can achieve high classification accuracy. This approach is especially practical in scenarios where obtaining large amounts of training data is difficult, particularly across diverse operating conditions.

##### Remark 5

The experiments have shown that the enhanced CNN-BiLSTM model effectively captures comprehensive and deeper sequential features from signals, resulting in improved classification accuracy. The HGWA algorithm has autonomously optimized training parameters, reducing the need for manual tuning and further enhancing accuracy. Moreover, model-based transfer learning has proven effective in addressing data scarcity, enabling high performance with limited training samples. This approach not only reduces sample requirements but also demonstrates strong generalization across various conditions, making it a robust solution for complex operating environments.

### Fault diagnosis using optimized CNN-BiLSTM on the JUN dataset

The JNU bearing dataset was gathered from the rolling bearing fault diagnosis test rig at Jiangnan University, specifically in a centrifugal fan system. The vibration frequency for this dataset was set to 50 kHz. The dataset includes measurements from four distinct fault conditions: normal operation, inner ring failure, outer ring failure, and ball bearing fault. Additionally, data were collected at three different rotational speeds. As shown in Table 9, the JNU dataset is categorized into 12 fault types, which are determined by various operational conditions and rotational speeds.

**Table 9:** Description of JNU bearing dataset.

Label	Fault mode	Rotating speed	Label	Fault mode	Rotating speed
0	Normal	600 rpm	6	Outer race fault 1	600 rpm
1	Normal	800 rpm	7	Outer race fault 2	800 rpm
2	Normal	1000 rpm	8	Outer race fault 3	1000 rpm
3	Inner race fault 1	600 rpm	9	Ball fault 1	600 rpm
4	Inner race fault 2	800 rpm	10	Ball fault 2	800 rpm
5	Inner race fault 3	1000 rpm	11	Ball fault 3	1000 rpm

**Fig. 10:**
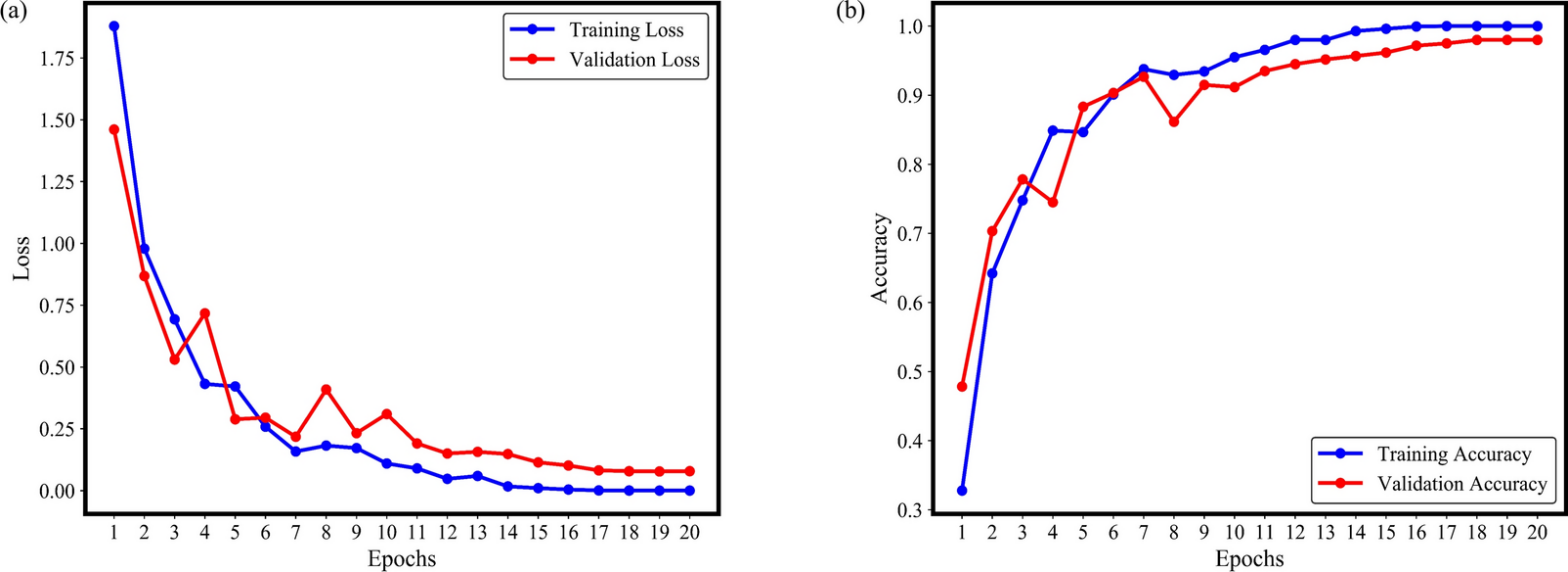
The convergence state and loss function on the JNU dataset: (**a**) Loss, (**b**) Accuracy.

**Fig. 11:**
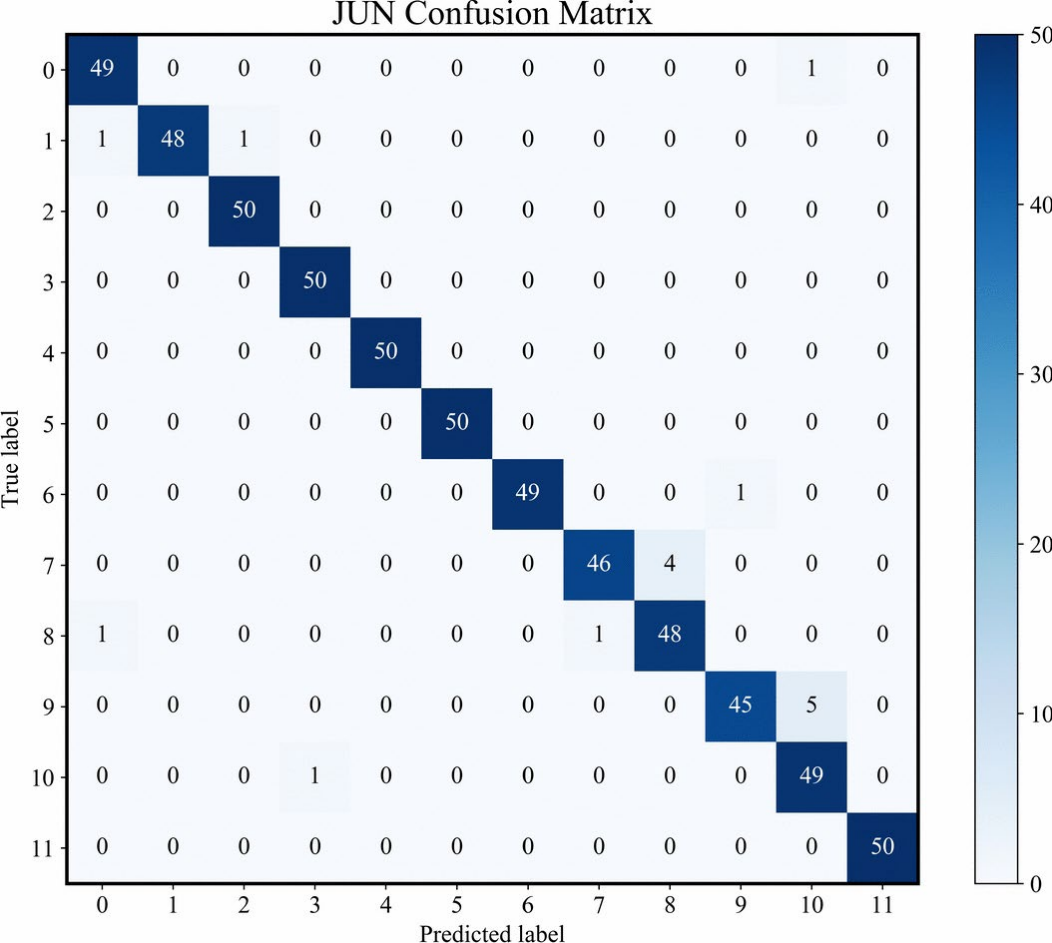
Confusion matrix of the testing set.

To assess the effectiveness of the HGWA-optimized CNN-BiLSTM model on the JNU dataset, an initial experiment was conducted. The preprocessing procedures and the range of hyperparameters explored were consistent with those used in the CWRU dataset experiments. Due to the increased complexity of the JNU training set, the number of training epochs has been adjusted to 20. Notably, previous experiments with this model were performed under single working conditions. To assess the HGWA-optimized CNN-BiLSTM model’s performance across multiple working conditions, this study has trained and optimized the model using samples from various conditions. This approach aims to validate the optimized model’s effectiveness under different scenarios. Emphasizing the transition from single-condition to multi-condition training is essential, as it highlights the model’s capability to manage more complex and variable data, demonstrating robustness and adaptability. The experimental results are presented in Figs. 10 and 11.

Figure 11 illustrates the variation in accuracy and loss for the HGWA-optimized CNN-BiLSTM model on the training and validation sets of the JNU dataset. After 20 epochs, the accuracy for both the training and validation sets stabilizes at approximately 98%. Similarly, the loss function converges and stabilizes as the number of epochs increases.

**Fig. 12:**
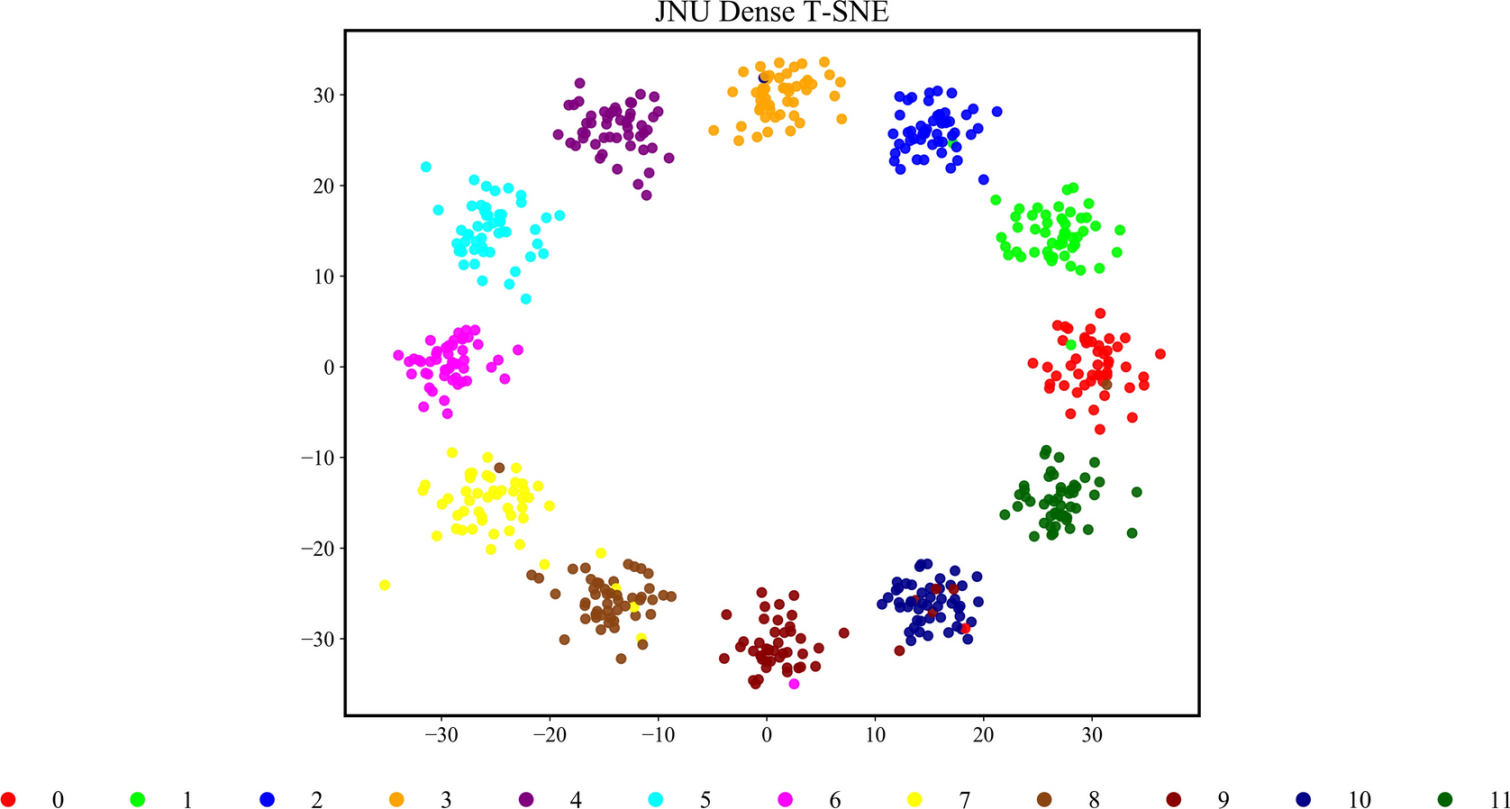
Classification of the testing set.

Figure 11 shows the confusion matrix for the HGWA-optimized CNN-BiLSTM model on the test set of the JNU dataset. The model achieves a high diagnostic accuracy for most fault signals, with an overall accuracy of 97.33%. Additionally, Fig. 12 visualizes the classification results of the test set using t-SNE. The clear boundaries between different faults further confirm the excellent diagnostic performance of the proposed model on the JNU dataset.

### Interpretability of the HGWA-CNN-BiLSTM framework

**Fig. 13:**
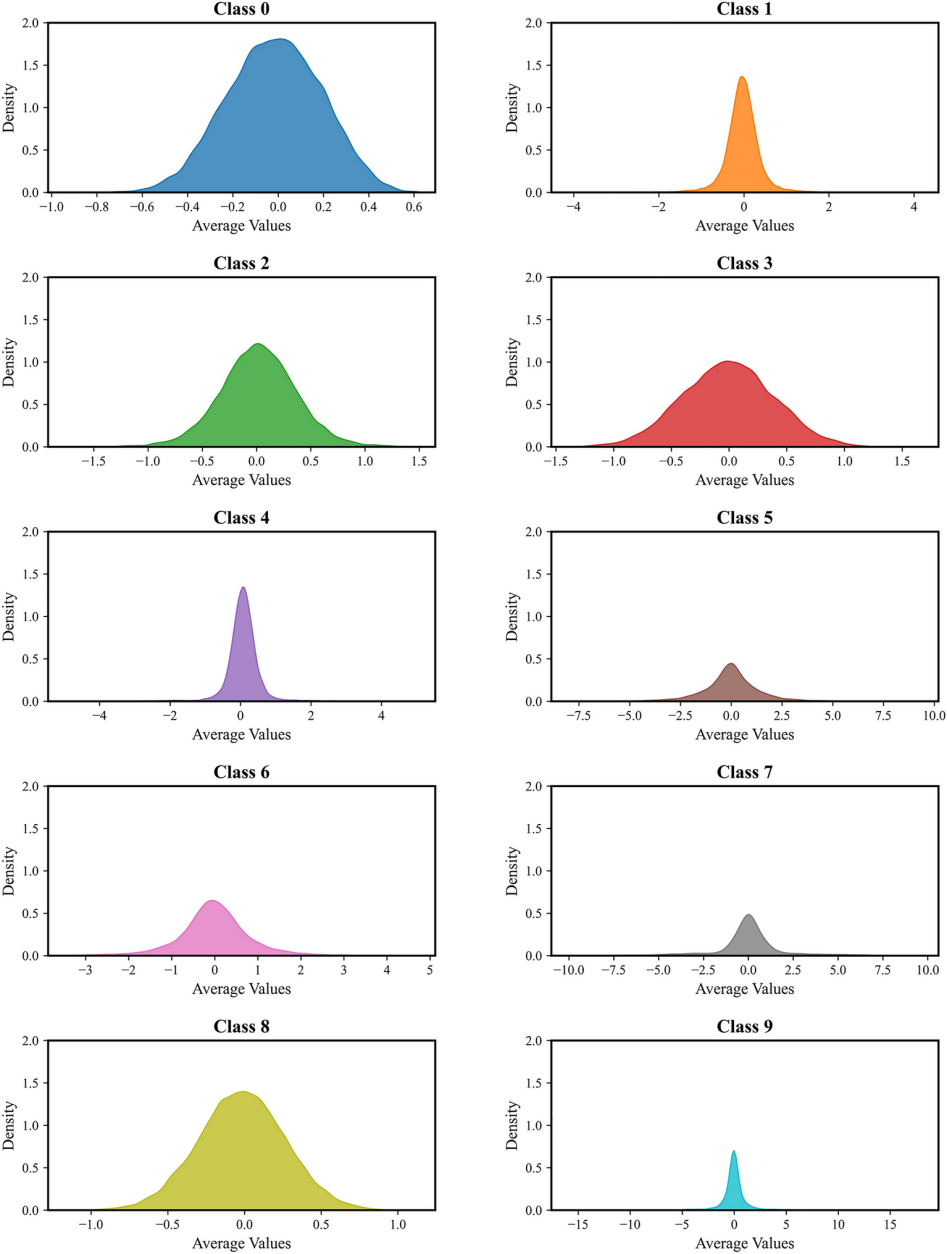
Ridgeline plot for 10 bearing failure data.

To effectively demonstrate the interpretability and reliability of the optimized CNN-BiLSTM framework’s diagnostic results, ridge plots have been used to analyze the fault classification outcomes. These plots offer an accurate representation of the mapping relationship between the inputs and outputs of the CNN-BiLSTM framework. Ridge plots are a data visualization technique that shows the distribution of continuous variables across different categories. This is accomplished by plotting multiple density estimation curves along the vertical axis, with each curve representing the distribution density of bearing data from different categories on the numerical axis. The curves are overlaid along the vertical axis, forming a ridge that displays the data distribution across the 10 bearing categories, as shown in Fig. 13.

Figure 13 has visualized the distribution characteristics of test samples from ten different bearing fault categories using ridgeline plots. Each category has exhibited a distinct density profile, reflecting variations in the underlying data distributions. Notably, Class 2 (outer race fault at the 6 o’clock position) and Class 8 (outer race fault at the 12 o’clock position) have demonstrated similar normal distribution patterns, both centered around a mean value of approximately 0.1. The peak density for Class 2 has reached 1.35, with a weight coefficient of 1.6, while Class 8 has peaked at 1.4, with a weight coefficient of 1.7. Despite the challenges posed by these similarities, the CNN-BILSTM-Net framework has achieved 100% accuracy in distinguishing these categories. The above analysis substantiates the reliability of the CNN-BILSTM-Net framework in diagnosing insulated bearing faults, further demonstrating its suitability for real-world industrial scenarios.

**Fig. 14:**
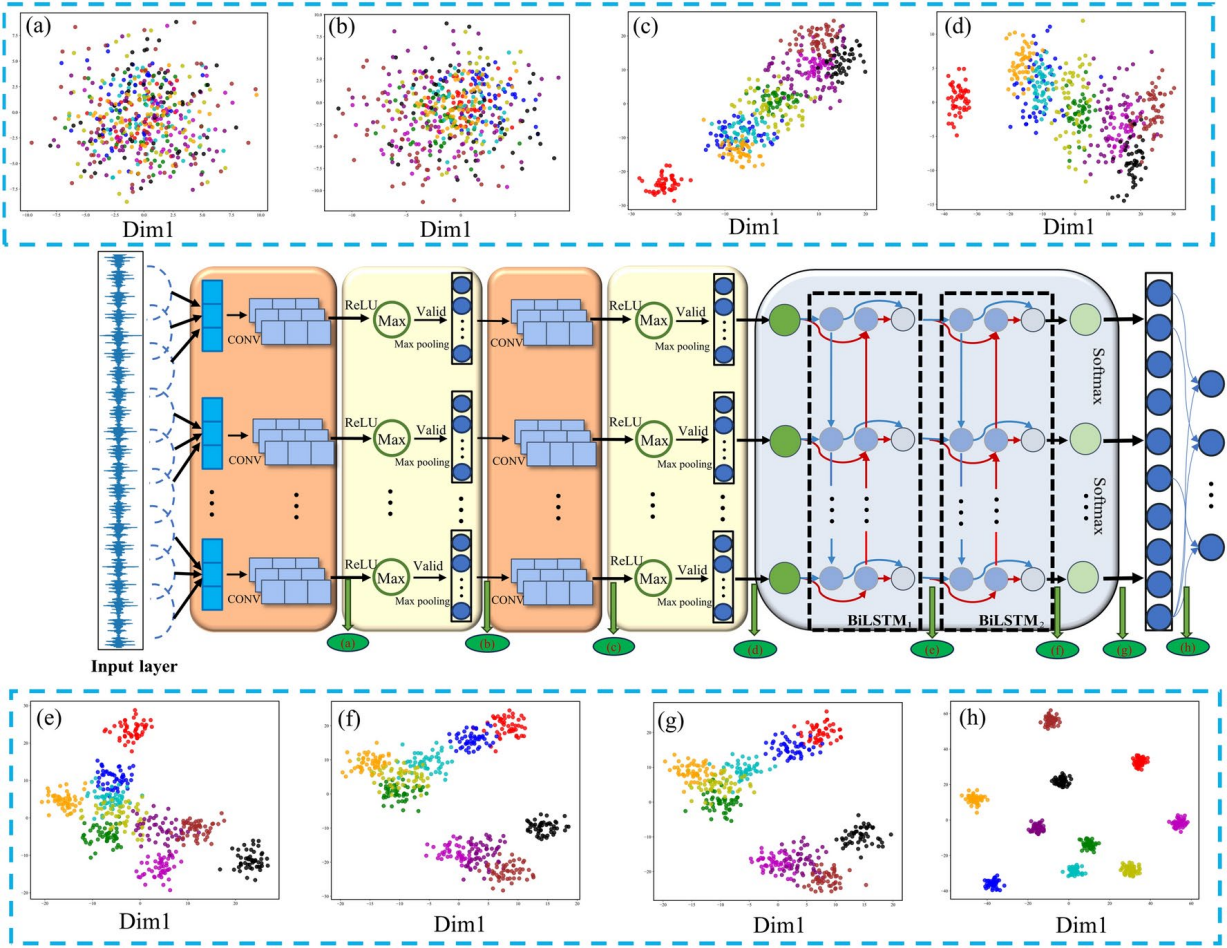
Visualization of the modules in the proposed CNN-BiLSTM framework.

To further illustrate the interpretability of the decision-making process of the CNN-BiLSTM framework for rolling bearing fault data, t-SNE dimensionality reduction has been introduced. This approach provides additional insights into the framework’s adaptive learning capabilities for fault features in rolling bearings. Using the SWRU bearing fault dataset D as an example, feature vectors representing ten different types of bearing faults have been extracted from each module. These feature vectors have been reduced to a two-dimensional space for visualization purposes. The results are presented in Fig. 14.

Figure 14 demonstrates the progressive refinement of feature boundaries among different bearing fault categories achieved by the optimized CNN-BiLSTM framework. As the framework depth increases, the separability between fault types becomes more distinct, with each category exhibiting well-defined boundaries, thereby enabling more precise fault differentiation.

Initially, when raw bearing signals are processed through the filters of the optimized convolutional kernel module, features from the ten fault types are scattered randomly across a two-dimensional space, with significant overlap between categories. This overlap arises from the inherent redundancy in the raw signals, resulting in a highly disordered and mixed data distribution prior to training.The application of the first convolution and pooling operations significantly enhances the separability of the extracted features. Features belonging to the same fault type begin to form cohesive clusters within defined regions, while those of different fault types diverge. This clustering effect is further enhanced after feature extraction by the coarse-grained module, underscoring the ability of the optimized convolutional module to capture key fault characteristics that reflect the operational conditions of the bearings.The BiLSTM module further reinforces the framework’s capacity to process and extract relevant bearing features. Following the aggregation module, the data exhibit strong intra-class cohesion and distinct inter-class separation, demonstrating the progressively enhanced non-linear representational capabilities of the CNN-BiLSTM framework with increasing depth. The globally extracted fault features are ultimately processed through the global average pooling layer and classified via the SoftMax function.

Experimental findings highlight substantial performance improvements achieved by the optimized CNN-BiLSTM model. By effectively extracting essential features from raw signals, the framework delivers highly accurate and reliable rolling bearing fault diagnosis. The improved separability and clustering of fault features with increasing model depth further validate the adaptive learning capacity of the framework in capturing and representing fault characteristics, confirming its efficacy in deep transfer fault diagnosis applications.

## Conclusion and future work

In industrial fault diagnosis, algorithms must strike a balance between real-time performance, model complexity, and computational efficiency, particularly in resource-constrained environments. While the HGWA-optimized CNN-BiLSTM model demonstrates high diagnostic accuracy, further refinement is required to enhance its real-time efficiency for practical deployment. Future research will focus on developing lightweight data preprocessing techniques, integrating efficient Transformer variants with CNNs for optimized time–frequency feature extraction, and advancing feature engineering through self-supervised learning and domain adaptation to improve robustness in noisy industrial settings. Despite its strong performance, challenges remain, particularly in diagnosing imbalanced fault categories, which requires techniques such as weighted loss functions and oversampling strategies. Additionally, computational bottlenecks under limited GPU memory must be addressed through optimization methods like model pruning and knowledge distillation, while small-sample scenarios call for the application of few-shot and transfer learning to improve generalization and reliability in real-world applications.

Future research will apply the proposed scheme to a range of fault detection and diagnosis problems, including wind turbine fault detection^55^, aircraft engine health monitoring^56^, smart grid fault diagnosis^57^, and unmanned aircraft system fault prediction^58^. To ensure broad applicability across these diverse domains, we will tackle key obstacles including data noise, scarcity, and variability by leveraging techniques such as transfer learning, domain adaptation, and architectural optimization. Moreover, emerging hierarchical fault classification approaches—capable of identifying fault types and assessing their severity through cognitive logic—will be further explored^59^. The interpretability of attention mechanisms within these frameworks will be enhanced by employing advanced methodologies, such as ensemble surrogate models and class activation mapping with proxy weighting^60,61^. Additionally, efforts will be directed towards strengthening the model’s resilience to noise, ensuring robust performance in real-world industrial environments.

## Data Availability

The datasets generated and/or analyzed during the current study are available upon request. Specifically: CASE Western Reserve University Bearing Data Center repository: Data is available from the corresponding author upon request. Jiangnan University dataset: The dataset is available from the corresponding author upon request via email at jiangliangthu@tsinghua.org.cn. Additionally, the experimental code and dataset preprocessing scripts are publicly available in our GitHub repository: https://github.com/ghfdcwklighqf/HGWA.
